# Supplementary material for: Lipidomics Reveals Elevated Plasmalogens in Women with Obesity Who Develop Preeclampsia
Source: J Clin Med. 2023 Apr 19;12(8):2970. doi: 10.3390/jcm12082970 (PMC10147017; doi:10.3390/jcm12082970)
Supplement: Supplementary file 1 [file jcm-12-02970-s001.zip › jcm-2272330-supplementary.pdf]

SUPPLEMENTAL TABLES

Table S1: Lipid differences by preeclampsia status across pregnancy[1]									
	Preeclampsia (n=33)			Uncomplicated Pregnancies (n=67)			p-values[2]		
	1st (s=29)	2nd (s=30)	3rd (s=20)	1st (s=52)	2nd (s=63)	3rd (s=65)	PE vs no PE	Trimester Change	PE x Trimester
**TG (mg/dL)**	106 (62.1)	133 (55.7)	206 (84.7)	89.5 (53.3)	133 (78.1)	190 (101)	0.64	**<0.0001**	0.97
**lnTG (mg/dL)**	4.52 (0.55)	4.81 (0.41)	5.25 (0.43)	4.36 (0.51)	4.78 (0.44)	5.14 (0.45)	0.38	**<0.0001**	0.62
**FFA (mEq/L)**	0.31 (0.19)	0.35 (0.20)	0.34 (0.12)	0.33 (0.22)	0.31 (0.18)	0.28 (0.18)	0.44	0.22	0.18
**lnFFA (mEq/L)**	-1.34 (0.59)	-1.23 (0.62)	-1.16 (0.39)	-1.33 (0.74)	-1.37 (0.63)	-1.43 (0.55)	0.21	0.69	0.18
**TC (mg/dL)**	165 (28.2)	207 (26.7)	217 (27.4)	166 (38.0)	196 (43.1)	221 (48.0)	0.72	**<0.0001**	0.47
**HDL (mg/dL)**	52.3 (14.2)	62.7 (16.7)	61.4 (17.1)	52.0 (12.0)	60.8 (15.2)	59.3 (14.2)	0.68	**<0.0001**	0.09
**LDL (mg/dL)**	91.8 (23.3)	117 (30.0)	115 (25.6)	95.8 (30.0)	110 (40.5)	124 (43.6)	0.89	**<0.0001**	0.97

[1]Values are represented as mean (standard deviation).
[2]P-values were calculated from linear mixed models with a random intercept for within-variation in each pregnancy. Natural log transformed variables are presented for any lipids that violated normality assumptions of model residuals and/or random intercepts.
Abbreviations: FFA, Free Fatty Acid; HDL, High-density Lipoprotein; LDL, Low-density Lipoprotein; s, Number of Biological Samples per Trimester; TC, Total Cholesterol; TG, Triglycerides.

Table S2: Lipid differences by fetal sex across pregnancy[1]									
	Male (n=53)			Female (n=47)			p-values[2]		
	1st (s=41)	2nd (s=51)	3rd (s=45)	1st (s=40)	2nd (s=42)	3rd (s=40)	Male vs Female	Trimester Change	Fetal Sex x Trimester
**TG (mg/dL)[3]**	102 (64.9)	138 (85.9)	211 (117)	88.7 (47.0)	128 (48.5)	175 (64.5)	0.13	**<0.0001**	0.15
**lnTG (mg/dL)**	4.45 (0.58)	4.79 (0.50)	5.23 (0.49)	4.37 (0.48)	4.79 (0.34)	5.10 (0.39)	0.34	**<0.0001**	0.66
**FFA (mEq/L)**	0.31 (0.21)	0.32 (0.19)	0.27 (0.14)	0.34 (0.20)	0.32 (0.18)	0.32 (0.19)	0.32	0.20	0.80
**lnFFA (mEq/L)**	-1.42 (0.73)	-1.32 (0.65)	-1.44 (0.50)	-1.25 (0.63)	-1.32 (0.61)	-1.29 (0.55)	0.27	0.62	0.90
**TC (mg/dL)**	167 (31.3)	199 (40.1)	224 (42.7)	164 (38.0)	201 (37.3)	215 (45.2)	0.40	**<0.0001**	0.97
**HDL (mg/dL)**	54.3 (14.1)	62.8 (16.2)	61.9 (15.5)	49.8 (10.8)	59.6 (15.0)	57.5 (13.9)	0.15	**<0.0001**	0.65
**LDL (mg/dL)**	92.8 (26.7)	110 (40.5)	122 (42.2)	96.0 (28.9)	116 (33.4)	122 (38.0)	0.95	**<0.0001**	0.42

[1]Values are represented as mean (standard deviation).
[2]P-values were calculated from linear mixed models with a random intercept for within-variation in each pregnancy. Natural log transformed variables are presented for any lipids that violated normality assumptions of model residuals and/or random intercepts.
Abbreviations: Refer to S1 Table.

Table S3: Lipid differences by preeclampsia status across pregnancy among women with female infants[1]									
	**Preeclampsia (n=14)**			**Uncomplicated Pregnancies (n=33)**			**p-values[2]**		
	**1st (s=14)**	**2nd (s=11)**	**3rd (s=8)**	**1st (s=26)**	**2nd (s=31)**	**3rd (s=32)**	**PE vs no PE**	**Trimester Change**	**PE x Trimester**
**TG (mg/dL)**	81.8 (31.1)	129 (38.1)	188 (68.2)	92.4 (53.9)	127 (52.3)	172 (64.3)	0.91	**<0.0001**	0.33
**lnTG (mg/dL)**	4.33 (0.40)	4.81 (0.32)	5.18 (0.35)	4.39 (0.52)	4.78 (0.35)	5.08 (0.40)	0.85	**<0.0001**	0.31
**FFA (mEq/L)**	0.33 (0.19)	0.33 (0.19)	0.34 (0.13)	0.35 (0.21)	0.31 (0.18)	0.32 (0.21)	0.93	0.46	0.76
**lnFFA(mEq/L)**	-1.26 (0.59)	-1.27 (0.58)	-1.17 (0.41)	-1.24 (0.67)	-1.34 (0.63)	-1.32 (0.58)	0.77	0.68	0.67
**TC (mg/dL)**	156 (21.8)	215 (24.0)	219 (17.1)	168 (44.3)	196 (40.1)	214 (50.0)	0.68	**<0.0001**	0.08
**HDL (mg/dL)**	47.6 (9.8)	61.0 (14.0)	58.7 (20.2)	51.0 (11.4)	59.1 (15.5)	57.2 (12.2)	0.94	**0.0003**	0.11
**LDL (mg/dL)**	92.0 (16.7)	129 (24.7)	122 (25.5)	98.2 (33.8)	111 (35.1)	122 (40.9)	0.63	**<0.0001**	0.27

[1]Values are represented as mean (standard deviation).
[2]P-values were calculated from linear mixed models with a random intercept for within-variation in each pregnancy. Natural log transformed variables are presented for any lipids that violated normality assumptions of model residuals and/or random intercepts.
Abbreviations: Refer to S1 Table.

Table S4: Lipid differences by preeclampsia status across pregnancy among women with male infants[1]									
	**Preeclampsia (n=19)**			**Uncomplicated Pregnancies (n=34)**			**p-values[2]**		
	**1st (s=15)**	**2nd (s=19)**	**3rd (s=12)**	**1st (s=26)**	**2nd (s=32)**	**3rd (s=33)**	**PE vs no PE**	**Trimester Change**	**PE x Trimester**
**TG (mg/dL)**	128 (75.4)	136 (64.6)	219 (95.1)	88.6 (53.6)	139 (97.4)	209 (125)	0.77	**<0.0001**	0.38
**lnTG (mg/dL)**	4.69 (0.62)	4.82 (0.46)	5.29 (0.49)	4.32 (0.51)	4.78 (0.52)	5.21 (0.49)	0.43	**<0.0001**	0.06
**FFA (mEq/L)**	0.29 (0.19)	0.36 (0.21)	0.34 (0.12)	0.32 (0.22)	0.30 (0.18)	0.25 (0.14)	0.20	0.31	0.12
**lnFFA(mEq/L)**	-1.40 (0.60)	-1.20 (0.66)	-1.16 (0.40)	-1.43 (0.80)	-1.40 (0.65)	-1.54 (0.50)	0.13	0.88	0.16
**TC (mg/dL)**	174 (31.4)	201 (27.4)	216 (33.3)	164 (31.2)	197 (46.4)	227 (45.8)	0.98	**<0.0001**	0.41
**HDL (mg/dL)**	56.6 (16.5)	63.6 (18.4)	63.2 (15.4)	53.0 (12.7)	62.3 (15.0)	61.4 (15.8)	0.67	**<0.0001**	0.44
**LDL (mg/dL)**	91.6 (28.7)	111 (31.3)	109 (25.3)	93.5 (26.1)	110 (45.8)	127 (47.0)	0.62	**<0.0001**	0.37

[1]Values are represented as mean (standard deviation).
[2]P-values were calculated from linear mixed models with a random intercept for within-variation in each pregnancy. Natural log transformed variables are presented for any lipids that violated normality assumptions of model residuals and/or random intercepts.
Abbreviations: Refer to S1 Table.

**Table S5: Lipid marker differences by race across pregnancy[1]**									
	**Black (n=66)**			**White (n=31)**			**p-values[2]**		
	**1st (s=52)**	**2nd (s=62)**	**3rd (s=58)**	**1st (s=27)**	**2nd (s=28)**	**3rd (s=26)**	**Black vs White**	**Trimester Change**	**Race x Trimester**
**TG (mg/dL)[3]**	78.0 (42.6)	113 (44.3)	153 (45.6)	122 (57.0)	180 (97.6)	282 (122)	**<0.0001**	**<0.0001**	**<0.0001**
**lnTG (mg/dL)**	4.24 (0.47)	4.67 (0.36)	4.98 (0.33)	4.70 (0.46)	5.09 (0.45)	5.56 (0.43)	**<0.0001**	**<0.0001**	0.46
**FFA (mEq/L)**	0.36 (0.21)	0.31 (0.19)	0.30 (0.17)	0.26 (0.19)	0.33 (0.19)	0.27 (0.16)	0.26	0.13	0.25
**lnFFA (mEq/L)**	-1.22 (0.67)	-1.35 (0.64)	-1.35 (0.53)	-1.55 (0.68)	-1.29 (0.64)	-1.43 (0.52)	0.26	0.50	0.20
**TC (mg/dL)**	160 (35.5)	193 (38.1)	208 (42.6)	174 (30.9)	213 (37.3)	246 (36.5)	**0.002**	**<0.0001**	**0.003**
**HDL (mg/dL)**	54.1 (13.7)	62.9 (16.2)	60.4 (14.2)	48.7 (10.1)	59.2 (13.4)	58.9 (16.6)	0.23	**<0.0001**	0.39
**LDL (mg/dL)**	90.2 (28.7)	107 (36.6)	117 (40.7)	101 (24.7)	121 (37.0)	135 (36.8)	0.06	**<0.0001**	0.22

[1]Values are represented as mean (standard deviation).
[2]P-values were calculated from linear mixed models with a random intercept for within-variation in each pregnancy. Natural log transformed variables are presented for any lipids that violated normality assumptions of model residuals and/or random intercepts.
Abbreviations: Refer to S1 Table.

**Table S6: Lipid differences by preeclampsia status across pregnancy among Black women[1]**									
	**Preeclampsia (n=22)**			**Uncomplicated Pregnancies (n=44)**			**p-values[2]**		
	**1st (s=19)**	**2nd (s=20)**	**3rd (s=14)**	**1st (s=33)**	**2nd (s=42)**	**3rd (s=44)**	**PE vs no PE**	**Trimester Change**	**PE x Trimester**
**TG (mg/dL)**	87.6 (44.4)	126 (46.4)	171 (54.7)	72.5 (41.2)	107 (42.4)	148 (41.4)	0.03	**<0.0001**	0.47
**FFA (mEq/L)**	0.37 (0.20)	0.35 (0.21)	0.34 (0.13)	0.35 (0.22)	0.29 (0.17)	0.28 (0.18)	0.21	0.10	0.60
**lnFFA(mEg/L)**	-1.16 (0.59)	-1.24 (0.65)	-1.15 (0.42)	-1.26 (0.72)	-1.40 (0.64)	-1.42 (0.55)	0.15	0.27	0.62
**TC (mg/dL)[3]**	161 (25.6)	202 (23.3)	209 (20.4)	159 (40.4)	188 (43.1)	208 (47.7)	0.57	**<0.0001**	0.50
**HDL (mg/dL)**	56.1 (15.8)	66.2 (17.0)	65.1 (15.9)	52.9 (12.5)	61.3 (15.8)	58.8 (13.5)	0.16	**<0.0001**	0.04
**LDL (mg/dL)**	87.2 (22.2)	110 (28.9)	110 (21.2)	91.9 (32.0)	106 (40.0)	119 (45.1)	0.68	**<0.0001**	0.72

[1]Values are represented as mean (standard deviation).
[2]P-values were calculated from linear mixed models with a random intercept for within-variation in each pregnancy. Natural log transformed variables are presented for any lipids that violated normality assumptions of model residuals and/or random intercepts.
Abbreviations: Refer to S1 Table.

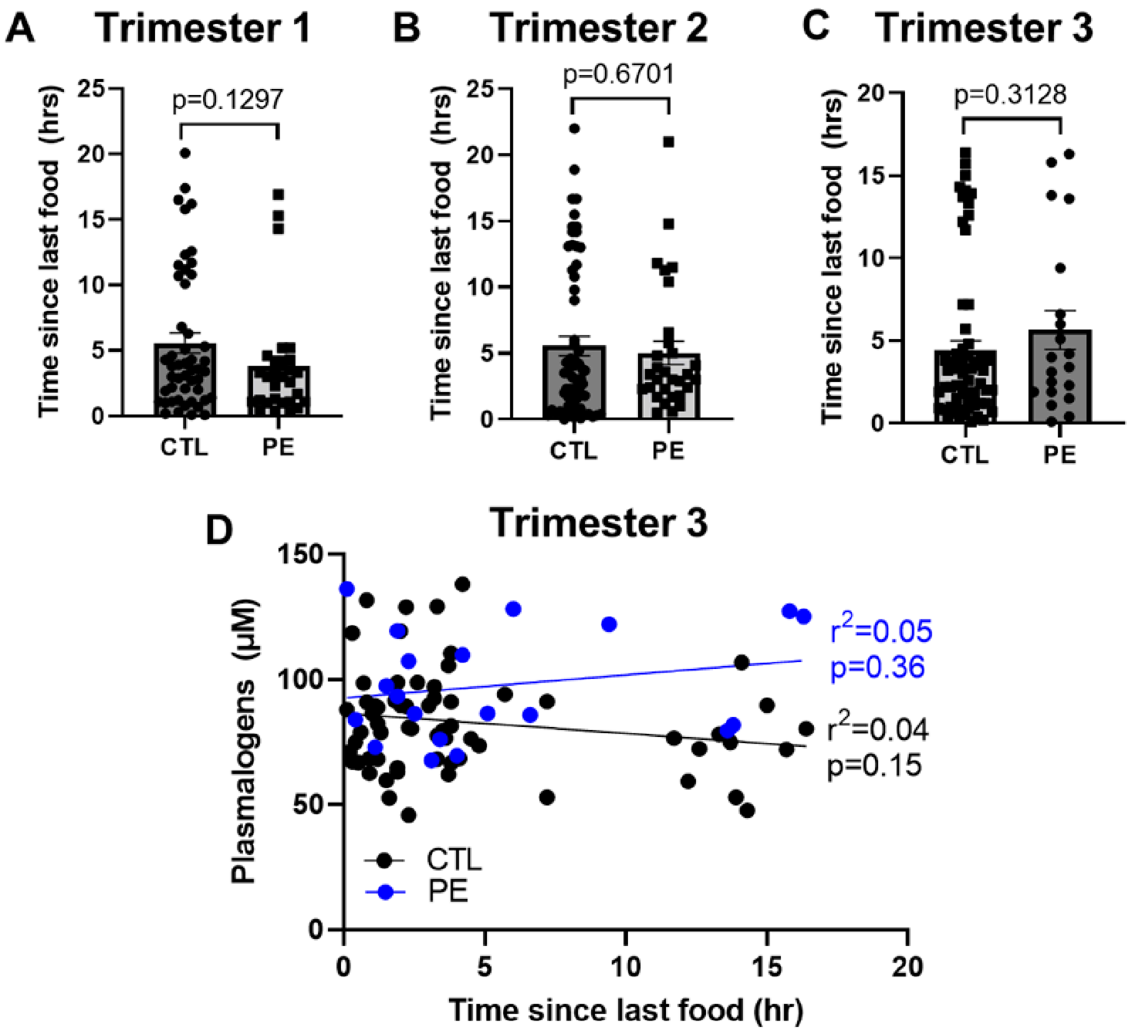

Supplemental Figure S1. Time since last food at sampling. A-C) Time since last food in CTL and PE groups at each trimester.Groups were compared by Student's t-test. D) Correlation between time since last food and plasmalogen levels in the third trimester in CTL and PE groups. R^2 and *p*-values were derived from linear regression.

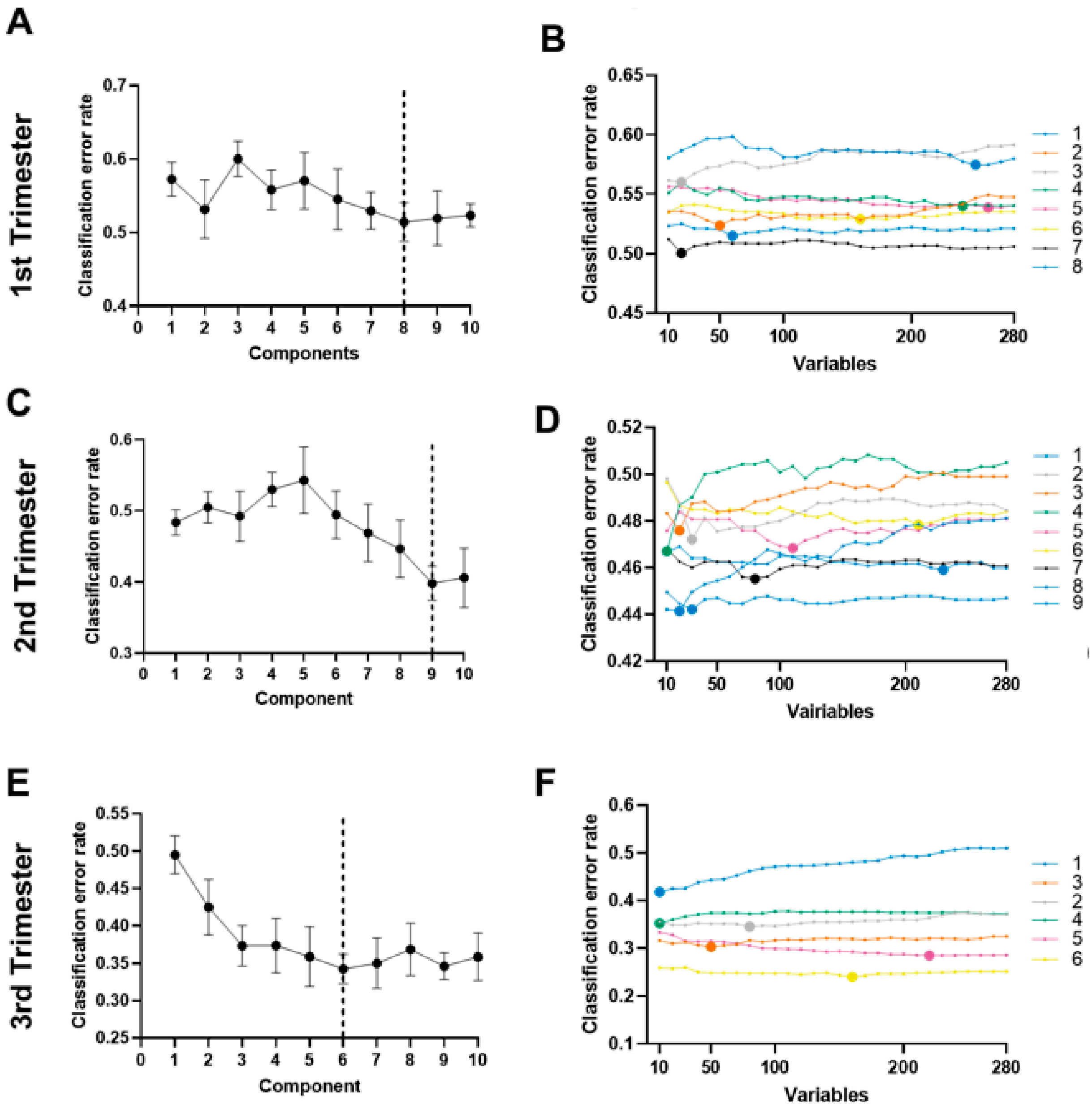

Supplemental Figure S2. sPLS-DA model tuning.A,C,E) Pregnancy outcome classification error rates as a function of the number of components used in the sPLS-DA model for lipidomic data from the A)first,B) second, or C) third trimester. The vertical line indicates the number of components that minimizes the error rate and is used in the final model.B,D,F) Classification error rate as a function of retained features for each component for the the sPLS-DA model for lipidomic data from each of the 3 trimesters.Large dots indicate the number of features that gave the lowest classification error and were used in thefinal model.

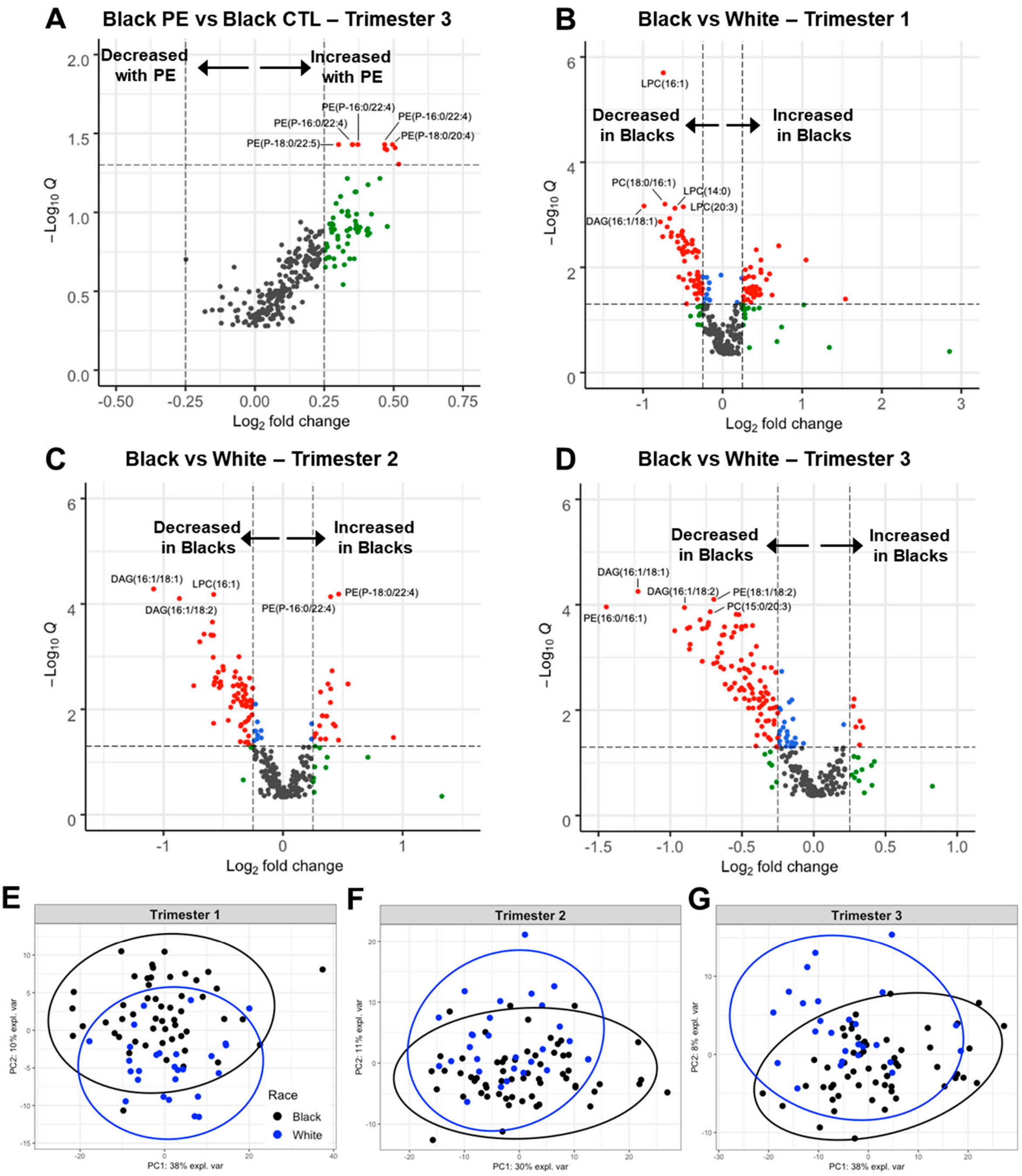

Supplemental Figure S3. Racial differences in plasma lipidomes during pregnancy.A-D)Volcano plots of 280 individuallipid species profiled in the indicated races and trimester.Red points are lipid species with log2FC >0.25 and q<0.1.Bluepoints have q<0.1.Green points have log2FC>0.25.Lipid species with q<0.1 are labeled.E-G)Lipidomic data from each trimester projected onto the first 2 principal components. Data points are colored by race.Ellipses indicate the 95% confidence interval of each group.

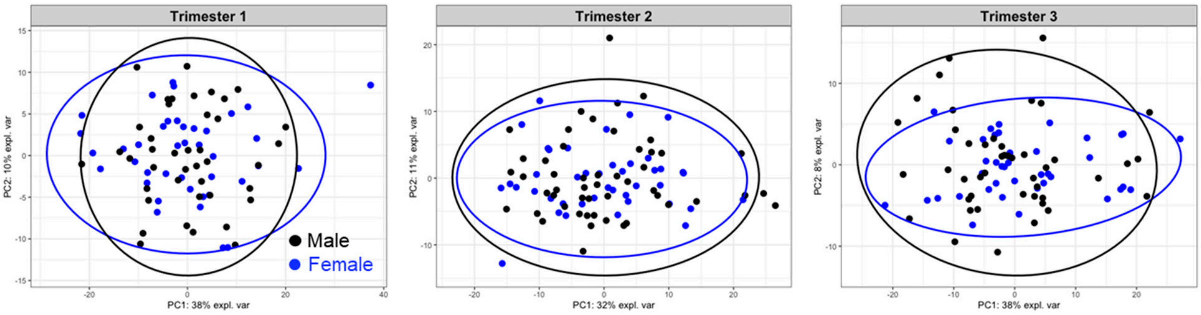

Supplemental Figure S4. Differences in plasma lipidomes based on fetal sex. Lipidomic data from each trimester projected onto the first 2 principal components. Data points are colored by fetal sex.Ellipses indicatethe 95% confidence interval of each group.
